# Severity and Outcomes of SARS-CoV-2 Reinfection Compared with Primary Infection: A Systematic Review and Meta-Analysis

**DOI:** 10.3390/ijerph20043335

**Published:** 2023-02-14

**Authors:** Jie Deng, Yirui Ma, Qiao Liu, Min Du, Min Liu, Jue Liu

**Affiliations:** 1 Department of Epidemiology and Biostatistics, School of Public Health, Peking University, Beijing 100191, China; 2 Institute for Global Health and Development, Peking University, Beijing 100871, China

**Keywords:** SARS-CoV-2, COVID-19, reinfection, severity, outcomes

## Abstract

Severe acute respiratory syndrome coronavirus 2 (SARS-CoV-2) reinfection has brought new challenges to the global prevention and control of coronavirus disease 2019 (COVID-19) pandemic; however, current studies suggest that there is still great uncertainty about the risk of severe COVID-19 and poor outcomes after SARS-CoV-2 reinfection. Random-effects inverse-variance models were used to evaluate the pooled prevalence (PP) and its 95% confidence interval (CI) of severity, outcomes and symptoms of reinfection. Random-effects were used to estimate the pooled odds ratios (OR) and its 95%CI of severity and outcomes between reinfections and primary infections. Nineteen studies involving a total of 34,375 cases of SARS-CoV-2 reinfection and 5,264,720 cases of SARS-CoV-2 primary infection were included in this meta-analysis. Among those SARS-CoV-2 reinfection cases, 41.77% (95%CI, 19.23–64.31%) were asymptomatic, and 51.83% (95%CI, 23.90–79.76%) were symptomatic, only 0.58% (95%CI, 0.031–1.14%) manifested as severe illness, and 0.04% (95%CI, 0.009–0.078%) manifested as critical illness. The PPs for SARS-CoV-2 reinfection-related hospitalization, admission to ICU, and death were, respectively, 15.48% (95%CI, 11.98–18.97%), 3.58% (95%CI, 0.39–6.77%), 2.96% (95%CI, 1.25–4.67%). Compared with SARS-CoV-2 primary infection cases, reinfection cases were more likely to present with mild illness (OR = 7.01, 95%CI, 5.83–8.44), and the risk of severe illness was reduced by 86% (OR = 0.14, 95%CI, 0.11–0.16). Primary infection provided some protection against reinfection and reduces the risk of symptomatic infection and severe illness. Reinfection did not contribute to extra risk of hospitalization, ICU, or death. It is suggested to scientifically understand the risk of reinfection of SARS-CoV-2, strengthen public health education, maintain healthy habits, and reduce the risk of reinfection.

## 1. Introduction

The global pandemic of severe acute respiratory syndrome coronavirus 2 (SARS-CoV-2) has brought great impacts and challenges to the global economy and human health. The COVID-19 epidemic has lasted for more than three years. According to the incomplete statistics reported to World Health Organization (WHO), as of February 2023, there have been more than 755 million confirmed cases of COVID-19, including more than 6.8 million deaths [1]. Existing studies suggest that SARS-CoV-2 constantly evolved and mutated during the epidemic process, and mutations at some sites might affect the ability of the virus to invade host cells, replicate and spread [2]. Some mutations might also resist antibodies produced after natural infection or vaccination, resulting in secondary or even multiple infections [2]. It seems that the dominant Omicron variants in the world currently have obvious immune escape characteristics, which could not only antagonize neutralizing antibodies, but could also escape the immune protection after natural infection, and might have a higher risk of reinfection [3]. The BA.2 subvariant of the Omicron variant has four additional mutations in the receptor binding domain (RBD), including S371F, T376A, D405N and R408S, which is more transmissible and immune escapable than the BA.1 subvariant, and which could breakthrough infect BA.1 survivors [4]. It is also suggested that BA.1-derived vaccine boosters may not achieve broad-spectrum protection against new Omicron variants [5]. Reinfection, commonly defined as a positive reverse transcription–polymerase chain reaction (RT-PCR) test ≥90 days from the primary infection, was rare in the early period of the coronavirus disease 2019 (COVID-19) pandemic [6]. In August 2020, the first observed SARS-CoV-2 reinfection case was reported in Hong Kong, China; a 33-year-old man was found to have asymptomatic reinfection after being discharged from hospital after recovery from the initial infection, with 142 days between two episodes [7]. Since then, an increasing number of studies have evaluated the probability and severity of reinfection after the primary infection. The results of a meta-analysis by Maria Elena Flacco et al. showed that the rate of SARS-CoV-2 reinfection was approximately 0.97%, and the risk of reinfection increased substantially over time, even reaching 3.31% in the first three months during the Omicron wave [8].

Benjamin Bowe et al. used the United States Department of Veterans Affairs’ national healthcare database to conduct a cohort study, suggesting that compared with non-reinfection, SARS-CoV-2 reinfection increased the risk of death by 117%, increased the risk of hospitalization by 232%, and increased the risk of having at least one sequela by 232% [9]. Furthermore, reinfection was positively associated with a higher risk of all-cause mortality, hospitalization, at least one sequela, and sequela in different physical systems, regardless of vaccination status [9]. Each infection increased the cumulative risk and affected the prognosis. The hazard ratio (HR) with at least one sequelae after the first infection was 1.35, increased to 2.11 after the second infection, and the HR could increase to 3.00 after three or more infections [4]. However, another nationwide study conducted in the United Kingdom (UK) showed that reinfection presented with milder symptoms and had a lower risk of COVID-19-related hospitalization and intensive care unit (ICU) admission compared with primary infection among those SARS-CoV-2 primary infection and reinfection cases collected from January 2020 to May 2021 [10]. A study conducted by Nežana Medić et al. in Serbia suggested that the rate of SARS-CoV-2 reinfection was about 5.49%, of which 99.17% of reinfection cases presented mild symptoms [11]. COVID-19-related hospitalization was not common, which was only 1.05%. The proportion of severe illness decreased from 5.47% among primary infections to 0.78% among reinfections, and the proportion of critically ill patients was only 0.05% [11].

Because of the differences in the definition of reinfection, epidemic period, follow-up time and other factors used in different studies, there is still great uncertainty about the risk of severe COVID-19 and poor outcomes after SARS-CoV-2 reinfection [8]. In addition, due to economic, policy, cultural, and geographical differences, the epidemic status, surveillance, and testing level of COVID-19 might vary among different countries. Most previous epidemiologic studies of SARS-CoV-2 reinfection were limited to explore the risk of reinfection. Omicron is still the dominant variant in the world currently. Compared with the wild-type virus, some Omicron subvariants have significantly enhanced immune escape ability and higher risk of reinfection, which has brought new challenges to the global prevention and control of COVID-19 pandemic [3,12]. Accurate assessment of the severity and outcomes of SARS-CoV-2 reinfection cases is essential for rational allocation of medical resources and optimization of vaccination strategies. Therefore, we aimed to review the data available to explore the severity and outcomes of SARS-CoV-2 reinfection and to conduct a systematic review and meta-analysis to provide a basis for the management of reinfection.

## 2. Methods

### 2.1. Search Strategy and Selection Criteria

We conducted a systematic search in PubMed, Embase, and Web of Science from database inception to 11 December 2022 without language restrictions by the following search terms: (COVID-19 OR SARS-CoV-2 OR coronavirus) AND (reinfection OR (repeat infection) OR (breakthrough infection)) AND (effect OR sequelae OR outcome OR prognosis OR (after effect)). We used EndNote X8.2 (Thomson Research Soft, Stanford, CA, USA) to manage records, screen, and exclude duplicates. This study was strictly performed according to the Preferred Reporting Items for Systematic Reviews and Meta-Analyses (PRISMA in the supplementary) [13]. This study was registered on PROSPERO (CRD42022382226).

The following studies were included: (1) studies that assessed the prevalence of severity of illness and outcome of SARS-CoV-2 reinfection; (2) studies that clarified the identification of SARS-CoV-2 reinfection, and the time interval between the two infections of the reinfection cases. The following studies were excluded: (1) irrelevant to SARS-CoV-2 reinfection; (2) insufficient data to calculate the prevalence of severity of illness and outcome of SARS-CoV-2 reinfection; (3) duplicate studies or overlapping participants; (4) reviews, editorials, conference papers, case report or series study, animal experiments and qualitative designs; (5) studies that did not clarify the identification of SARS-CoV-2 reinfection; and (6) studies that did not clarify the time interval between the two infections of the reinfection cases.

Studies were identified by two investigators (D.J. and M.Y.R.) independently following the criteria above, while discrepancies were solved by consensus or with a third investigator (L.Q.).

### 2.2. Data Extraction

The following data were extracted from the included studies: (1) basic information of the studies, including the first author, article type, study design, publication time, location where the study was conducted; (2) characteristics of the cases, including identification of SARS-CoV-2 reinfection, time range for inclusion of the primary infection and reinfection, time interval between the two infections, sample size, age, sex ratio, body mass index (BMI), comorbidity, smoking status, vaccination status, and the variant wave of the primary infection and reinfection. (3) severity of SARS-CoV-2 primary infection and reinfection: the proportion or the cases of asymptomatic infection, symptomatic infection, mild illness, severe illness, and critical illness; (4) outcome of SARS-CoV-2 primary infection and reinfection: the proportion or the cases of hospitalization, admission to ICU, death and so on; and (5) symptoms of symptomatic SARS-CoV-2 reinfection.

Data extraction and determination of information eligibility were conducted by two investigators (D.J. and M.Y.R.) independently following the criteria above, while discrepancies were solved by consensus or with a third investigator (L.Q.).

### 2.3. Quality Assessment and Risk of Bias 

We used the Newcastle–Ottawa quality assessment scale to evaluate the risk of bias of the included cohort studies and case-control studies. Cohort studies and case-control studies were classified as having low (≥7 stars), moderate (5–6 stars), and high risk of bias (≤4 stars) with an overall quality score of 9 stars. We used Egger’s test to evaluate the publication bias of the consequences with more than 4 data sources; *p* > 0.05 was considered as no publication bias.

Quality assessment was conducted by two investigators (D.J. and M.Y.R.) independently, while discrepancies were solved by consensus or with a third investigator (L.Q.).

### 2.4. Data Synthesis and Statistical Analysis

We performed a meta-analysis to estimate the pooled prevalence (PP) and its 95% confidence interval (CI) of: (1) severity of illness and outcomes of SARS-CoV-2 primary infection and reinfection; and (2) symptoms of symptomatic SARS-CoV-2 reinfection. In addition, we estimated the odd ratio (OR) and its 95% CI of severity of illness and outcomes of SARS-CoV-2 reinfection compared with primary reinfection and conducted a subgroup analysis of outcomes of SARS-CoV-2 reinfection and primary infection by time interval between two infections.

Random-effects or fixed-effects models were used to pool the rates and adjusted estimates across studies separately, based on the heterogeneity between estimates (I²). Fixed-effects models would be used if I² ≤ 50%, which represents low to moderate heterogeneity, and random-effects models would be used if I² ≥ 50%, representing substantial heterogeneity. The D-L method was used to estimate the tau square in the case of random-effects models. All analyses used Stata version 16.0 (Stata Corp, College Station, TX, USA).

## 3. Results

### 3.1. Basic Characteristics

**Figure 1:**
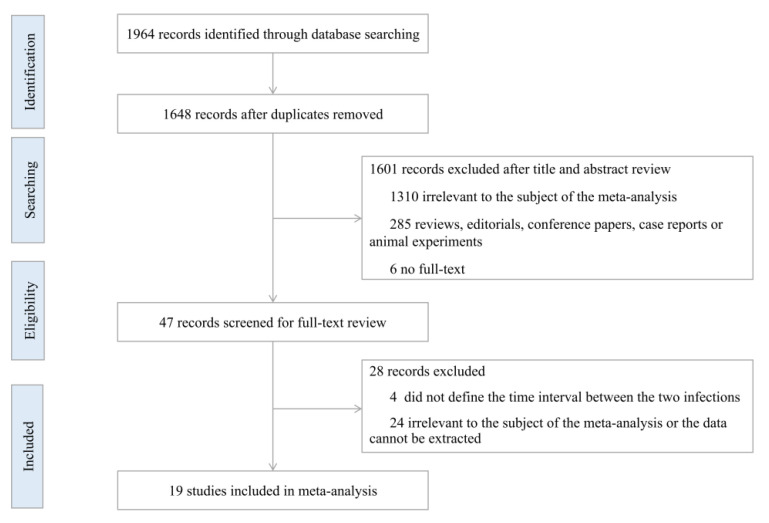
Flowchart for study selection.

In the original literature retrieval, a total of 1964 potential articles were identified up to 11 December 2022 through a database search (262 in PubMed, 195 in Embase, 1507 in Web of Science); 316 duplicates were excluded. After reviewing the titles and abstracts among the remaining 1648 articles, we excluded 1601 articles according to the inclusion and exclusion criteria. Among the remaining 47 studies under full-text reading, 28 studies were excluded. Eventually, 19 articles were included in this meta-analysis and systematic review based on the inclusion criteria [10,11,14,15,16,17,18,19,20,21,22,23,24,25,26,27,28,29,30] The literature retrieval flow chart is shown in Figure 1.

Of the 19 articles included, most studies were conducted in the United States of America (USA, n = 4), followed by Italy (n = 2), Turkey (n = 2) and England (n = 2), and the rest from Austria, France, Israel, Kuwait, Spain, Serbia, India, Qatar and other countries. In most of the included studies (n = 15), SARS-CoV-2 reinfection was defined as a laboratory-confirmed SARS-CoV-2-positive test on a polymerase chain reaction (PCR) or antigen test at least 90 days after the initial confirmed positive test. The included studies explored the illness severity and outcomes of SARS-CoV-2 reinfection, involving a total of 34,375 cases of SARS-CoV-2 reinfection and 5,264,720 cases of SARS-CoV-2 primary infection, while the time interval between two infections of most included studies was more than 90 days. The characteristics of the included studies are shown in Supplementary Materials Table S1.

### 3.2. Pooled Prevalence (PP) of Severity and Outcomes of SARS-CoV-2 Reinfection

We estimated the PPs of illness severity and outcomes of SARS-CoV-2 reinfection and primary infection cases. Among those SARS-CoV-2 reinfection cases, 41.77% (95%CI, 19.23–64.31%) were asymptomatic, and 51.83% (95%CI, 23.90–79.76%) presented with symptomatic infection. The severity of 65.55% reinfection cases was mild; only 0.58% (95%CI, 0.031–1.14%) manifested as severe illness and 0.04% (95%CI, 0.009–0.078%) manifested as critical illness. In terms of outcomes, the PPs for SARS-CoV-2 reinfection-related hospitalization, admission to ICU, and death were, respectively, 15.48% (95%CI, 11.98–18.97%), 3.58% (95%CI, 0.39–6.77%), and 2.96% (95%CI, 1.25–4.67%). Among SARS-CoV-2 primary infection cases, 85.45% (95%CI, 83.87–87.03%) were symptomatic infection, 94.48% (95%CI, 94.39–94.57) presented with mild illness, 5.47% (5.38–5.56%) presented with severe illness, and only 0.05% (0.041–0.059%) presented with critical illness. The PPs for SARS-CoV-2-related hospitalization and death were, respectively, 9.51% (95%CI, 2.711–6.31%) and 8.58% (95%CI, 6.78–10.38%). More analysis results are shown in Table 1.

### 3.3. Pooled Prevalence (PP) of Symptoms among Symptomatic SARS-CoV-2 Reinfection Cases

We estimated the PPs of a total of nine symptoms among those symptomatic SARS-CoV-2 infection cases. The most common symptom was fever (PP = 35.46%, 95%CI, 24.92–46.00%), followed by cough (PP = 28.04%, 95%CI, 18.09–37.99%), fatigue (PP = 24.33%, 95%CI, 12.33–36.32%), diarrhea (PP = 12.90%, 95%CI, 1.10–24.70%), nausea/vomiting (PP = 12.19%, 95%CI, 3.03–21.35%), and sore throat (PP = 7.93%, 95%CI, 1.11–14.74%). More analysis results are shown in Table 2.

### 3.4. Comparison of Severity and Outcomes of SARS-CoV-2 Reinfection and Primary Infection

We compared the severity and outcomes of SARS-CoV-2 infection between reinfection and primary infection. As shown in Table 3, the risk of symptomatic infection among reinfection cases was only 0.01 of that among primary infection cases (OR = 0.01, 95%CI, 0.007–0.011). Compared with SARS-CoV-2 primary infection cases, reinfection cases were more likely to present with mild illness (OR = 7.01, 95%CI, 5.83–8.44), while the risk of severe illness was reduced by 86% (OR = 0.14, 95%CI, 0.11–0.16). There were no significant differences in other severity and outcomes between SARS-CoV-2 reinfection and primary infection cases. We also conducted a subgroup analysis of outcomes of SARS-CoV-2 reinfection and primary infection by time interval between two infections; the results suggested that if the time interval ≥ 90 days, reinfections had a lower risk of COVID-19-related hospitalization compared with primary infections (OR=0.33, 95%CI, 0.11-1.00). However, if the time interval was only ≥ 28 days, it seems that reinfections would have a higher risk of COVID-19-related hospitalization (OR = 11.82, 95%CI, 4.36–32.03), as seen in Supplementary Materials Table S2.

### 3.5. Quality Evaluation and Publication Bias

We evaluated the quality of the included cohort studies and case-control studies according to the Newcastle–Ottawa quality assessment scale; most of them were of good quality and had a low risk of bias (n = 15, ≥7 stars). The remaining four studies were of moderate quality and had moderate risk of bias (6 stars), as shown in Supplementary Table S1. We evaluated the publication bias of the consequences with more than four data sources using Egger’s test. There was publication bias in the PP of admission to ICU after SARS-CoV-2 reinfection and the PP of death after SARS-CoV-2 primary infection (*p* < 0.05) and no publication bias in the remaining consequences (*p* > 0.05).

## 4. Discussion

COVID-19 is spreading rapidly around the world, with a large number of new infections. While many patients have gradually recovered, there is growing evidence that reinfection is possible after previous infections. At present, more and more scholars have begun to pay attention to the disease severity and outcomes of SARS-CoV-2 reinfection. However, due to the differences in the definition of reinfection, epidemic period, follow-up time and other factors in different studies, the severity and outcomes after the reinfection of SARS-CoV-2 are still uncertain [8]. This systematic review and meta-analysis of 19 studies, involving 34,375 cases after SARS-CoV-2 primary infection and 5,264,720 after reinfection, provided PP of SARS-CoV-2 reinfection severity and outcomes and OR of severity and outcomes of reinfection compared with primary infection.

Available data showed that patients after SARS-CoV-2 reinfection usually reported clinical symptoms, with PP of 51.83%. Among the nine outcomes evaluated in this study, fever, cough and fatigue were the most common, which is consistent with the results of the previous study [31]. Rubaid Azhar Dhillon et al. found that reinfection cases had a higher frequency of difficulty breathing and fatigue and that the frequency of other symptoms was not statistically different from that after a primary infection [31], which may be related to the fact that the respiratory tract is the first target invaded by SARS-CoV-2. Cough and dyspnea are often associated with damage to the upper respiratory tract. Previous studies have shown that COVID-19 infection mainly affects the upper respiratory tract, followed by the lower respiratory tract [32], and viral load in the upper respiratory tract is related to the severity and outcomes of COVID-19 [32].

Available data showed that the pooled mortality rate of SARS-CoV-2 reinfection was 2.95%, which was similar to the results of a previous study [10]. There is a wide age distribution of reinfection in the population, and older people face a higher risk of adverse outcomes. Jillian N. Armstrong found that the mortality rate of people with median age 75 years old after SARS-CoV-2 reinfection was higher, up to 12.8% [16]. Benjamin Bowe found that compared with non-reinfection, SARS-CoV-2 reinfection contributed additional risk of death by 117%, hospitalization by 232%, and at least one sequela by 232% in people aged more than 60 years [9]. This may be related to low immunity, complications and basic diseases in the elderly [10]. Therefore, prevention of SARS-CoV-2 reinfection is very important for the elderly.

Available data showed that reinfections had a lower risk of symptomatic infection and severe illness than primary infections and were more likely to be mild, which is consistent with previous findings [18] and may be related to durable immunity from primary immunization and vaccination. Qi Chen found that in the unvaccinated population, the protection of naturally acquired antibodies against reinfection was 84% [33]. A study from Israel found that at least one dose of BNT162b2 vaccine was 82% effective in preventing reinfection in previously infected people aged 16-64 and was 60% in people aged 65 and older, and that the effectiveness of one dose was not significantly different from that of two doses [34]. Snežana Medić et al. found that booster shots may reduce the risk of reinfection modestly [11]. The subgroup analysis results of this study showed that reinfections had a lower risk of COVID-19-related hospitalization compared with primary infections with the time interval ≥90 days, which might suggest a mild long-term consequence of SARS-CoV-2 reinfection. On the one hand, this might be related to the persistent high prevalence of antibodies against SARS-CoV-2, which has been found in previous studies to bring at least 6–8 months of immunity to most people [35]. On the other hand, it might also be related to the mild symptoms caused by reinfection; therefore, more patients chose self-treatment such as resting at home or self-medicating. Evidence showed that the reinfection rate of SARS-CoV-2 varied greatly between countries and territories, with 1.08% in America, 0.77% in Asia and 0.63% in Europe [8], which might be related to different economic levels, sanitary conditions, public awareness of SARS-CoV-2 prevention, policies and vaccination coverage.

At present, studies on the reinfection rate of different SARS-CoV-2 VOCs are limited, and the results of previous studies are not completely consistent. A cohort study from Brazil showed that the SARS-CoV-2 reinfection rate in health workers was 4.3% during the Omicron period, which was higher than the period before Omicron (0.8%) [36]. A meta-analysis including 91 studies showed that the pooled rate of reinfection was highest in the Omicron-predominant period (3.31%), followed by the Delta- (1.25%) and Alpha- (0.57%) predominant periods [8]. The protection of primary infection against reinfection with different variants of SARS-CoV-2 was different, which was 90.2% against the Alpha variant, 85.7% against the Beta variant, and 92.0% against the Delta variant [37]. The protection against Omicron was the lowest (56.0%), which may be related to its strong immune escape ability [37]. People infected with SARS-CoV-2 can develop cross-immunity against different variants for a certain time, but the protection against re-infection with the same strain is significantly higher. Heba N. Altarawneh found that if infected by the SARS-CoV-2 strain prior to Omicron, the effectiveness of the reinfection of BA.4 and BA.5 was only 27.7%, and if the primary infection was the Omicron strain, the effectiveness of the reinfection of BA.4 and BA.5 was 78.0% [38]. XBB is a recombinant of Omicron variant BA.2.10.1 and BA.2.75 sublineages with enhanced immune escape ability, which was first identified in India in August 2022. Since then, XBB has spread rapidly and has become the dominant strain in Singapore, Malaysia, and some European and American countries, and has gradually evolved into XBB.1, XBB.1.1, XBB.1.5, XBB.2 and other Omicron variant subbranches [39,40,41]. Due to late onset, the severity and outcomes of reinfection with the XBB strain is unclear. There is, however, early evidence pointing at a higher reinfection risk, as compared to other circulating Omicron sublineages [42]. Cases of reinfection were primarily limited to those with initial infection in the pre-Omicron period [42]. Attention should be paid to the immune evasion of emerging XBB strains and the increased risk of reinfection, which might bring new difficulties and challenges to the prevention and control of the COVID-19 pandemic. More studies of reinfection with the Omicron strain and its endemic subtype variants are needed in the future.

For those who have recovered from the primary infection of SARS-CoV-2, the best way to prevent reinfection is to take good protective measures, including wearing masks, paying attention to hand hygiene, taking good rest, frequent ventilation, and maintaining social distancing. Especially for the elderly, people with low immunity, and patients who have just recovered, they should pay attention to keeping warm, minimizing gatherings, ensuring good hygiene habits, as well as pay attention to maintaining a nutritious diet, enhancing immunity, and minimizing the risk of reinfection.

There are some limitations in this study. Firstly, publication bias existed in some evaluated consequences, and it should also be considered in other ones since the data sources were limited. Secondly, there was high heterogeneity in the PPs of severity of illness and outcomes of SARS-CoV-2 reinfection, which may be related to the characteristics of the population such as gender, age, countries and regions, underlying diseases, variants, time interval between the primary infection and reinfection of SARS-CoV-2, and so on. However, due to limited data, subgroup analysis could only be performed by time interval. Thirdly, patients might report the long-term clinical symptoms of the first infection as the symptoms of the reinfection due to the inability to distinguish between the two infections; therefore, that potential bias should be considered.

## 5. Conclusions

Primary infection provides some protection against reinfection and reduces the risk of symptomatic infection and severe illness. Reinfection did not contribute to extra risk of hospitalization, ICU, or death. It is suggested to scientifically understand the risk of reinfection of SARS-CoV-2, strengthen public health education, maintain healthy habits, and reduce the risk of reinfection. 

## Figures and Tables

**Table 1:** Pooled prevalence (PP) of severity of illness and outcomes of SARS-CoV-2 reinfection and primary infection.

Infection Status	Reinfection						Primary Infection					
Consequences	Data Source	Patients n/N	PP (%)	95%CI (%)	*p* Value	I^2^ (%)	Data Source	Patients n/N	PP (%)	95%CI (%)	*p* Value	I^2^ (%)
**Severity of illness**												
Asymptomatic infection	2, 5, 7, 8, 10, 11, 15, 18	1326/4786	41.77	19.23–64.31	<0.05	99.7	13, 18, 20	237,198/655,381	19.61	−3.70–42.92	>0.05	100
Symptomatic infection	2, 3, 4, 5, 10, 11, 12, 18	1519/4190	51.83	23.90–79.76	<0.05	99.7	13	1638/1917	85.45	83.87–87.03	<0.05	–
Mild illness	7, 9	13,687/13,821	65.65	−1.12–132.41	>0.05	98.4	12	237,243/251,104	94.48	94.39–94.57	<0.05	–
Severe illness	7, 9, 14	116/15,125	0.58	0.031–1.14	<0.05	83.3	12	13,735/251,104	5.47	5.38–5.56	<0.05	–
Critical illness	9, 14	6/15,096	0.04	0.009–0.078	<0.05	-	12	126/251,104	0.05	0.041–0.059	<0.05	–
**Outcomes**												
Hospitalization	1, 2, 3, 4, 5, 6, 7, 8, 9, 12, 13, 16, 17, 18, 19	1764/29,692	15.48	11.98–18.97	<0.05	99	12, 18, 21, 24	33,124/325,982	9.51	2.7116.31	<0.05	99.9
Admission to ICU	2, 5, 15, 16, 19	30/2869	3.58	0.39–6.77	<0.05	86.3	21, 24	1620/59,803	1.48	−1.02–3.98	>0.05	99.6
Death	1, 2, 4, 5, 6, 8, 9, 12, 14, 15, 16, 17	453/33,501	2.96	1.25–4.67	<0.05	97.7	1, 13, 17, 20, 21, 22, 23	5999/800,417	8.58	6.78–10.38	<0.05	99.9
Need for mechanical ventilation	5, 18	3/74	3.79	−0.55–8.14	>0.05	0	-	-	-	-	-	-

**Table 2:** Pooled prevalence (PP) of symptoms of symptomatic SARS-CoV-2 reinfection.

Symptoms	Data Source	Patients n/N	PP (%)	95%CI (%)	*p* Value	I^2^ (%)
Fever	3, 7, 18	28/78	35.46	24.92–46.00	<0.05	0
Cough	3, 7, 18	22/78	28.04	18.09–37.99	<0.05	0
Shortness of breath	3, 7	16/60	25.4	−5.52–56.33	>0.05	88.9
Fatigue	3, 18	12/49	24.33	12.33–36.32	<0.05	0
Diarrhea	3, 7	4/60	12.90	1.10–24.70	<0.05	-
Nausea/vomiting	3, 18	6/49	12.19	3.03–21.35	<0.05	0
Myalgia	3, 7	6/60	8.52	−3.66–20.70	>0.05	65.7
Headache	3, 7	6/60	8.52	−3.66–20.70	>0.05	65.7
Sore throat	3, 7	5/60	7.93	1.11–14.74	<0.05	0

**Table 3:** Comparison of severity of illness and outcomes of SARS-CoV-2 reinfection and primary infection.

Consequences	Data Source	Reinfection n/N	Primary Infection n/N	OR	95%CI (%)	*p* Value	I^2^ (%)
**Severity of illness**							
Asymptomatic infection	10, 15	1060/4030	236,093/640,306	0.34	0.013–8.65	>0.05	99.5
Symptomatic infection	14	94/1917	1638/1917	0.01	0.007–0.011	<0.05	-
Mild illness	9	13,678/13,792	237,243/251,104	7.01	5.83–8.44	<0.05	-
Severe illness	9	108/13,792	13,735/251,104	0.14	0.11–0.16	<0.05	-
Critical illness	9	6/13,792	126/251,104	0.87	0.38–1.97	>0.05	-
**Outcomes**							
Hospitalization	9, 13, 16, 19	193/14,237	33,124/325,982	0.95	0.23–3.92	>0.05	97.4
Admission to ICU	16, 19	21/440	1620/59,803	14.11	0.068–2909.82	>0.05	97.5
Death	1, 12, 15, 16, 17	30/2971	5162/792,331	0.89	0.36–2.23	>0.05	67.7

## Data Availability

Data are available from the corresponding author by request.

## Supplementary Materials

The following supporting information can be downloaded at: https://www.mdpi.com/article/10.3390/ijerph20043335/s1, PRISMA_2020_checklist.pdf; Supplementary Table S1. Characteristics of the included studies; Supplementary Table S2. Subgroup analysis of comparison of outcomes of SARS-CoV-2 reinfection and primary infection by time interval between two infections.

## Abbreviations

COVID-19	coronavirus disease 2019
SARS-CoV-2	severe acute respiratory syndrome coronavirus 2
RBD	receptor binding domain
RT-PCR	reverse transcription–polymerase chain reaction
UK	United Kingdom
ICU	intensive care unit
WHO	World Health Organization
PRISMA	Preferred Reporting Items for Systematic Reviews and Meta-Analyses
PP	pooled prevalence
OR	odds ratio
CI	confidence interval
HR	hazard ratio
