# Supplementary material for: Severity and Outcomes of SARS-CoV-2 Reinfection Compared with Primary Infection: A Systematic Review and Meta-Analysis
Source: Int J Environ Res Public Health. 2023 Feb 14;20(4):3335. doi: 10.3390/ijerph20043335 (PMC9961977; doi:10.3390/ijerph20043335)
Supplement: Supplementary file 1 [file ijerph-20-03335-s001.zip › supplementary Table S2.pdf]

Supplementary table s2. Subgroup analysis of comparison of outcomes of SARS-CoV-2 reinfection and primary infection by time interval between two infections

Consequences	Number of data source	Reinfection n/N	Primary infection n/N	OR	95%CI(%)	P-value	I^2(%)	P-Heterogeneity
Hospitalization								
≥ 90 days	3	187/14,205	33,105/324,990	0.33	0.11-1.00	<0.05	95.3	<0.05
≥ 28 days	1	6/32	19/992	11.82	4.36-32.03	<0.05	-	-
Admission to ICU								
≥ 90 days	1	11/408	1618/58,811	0.98	0.54-1.79	>0.05	-	-
≥ 28 days	1	10/32	2/992	225	46.54-1087.86	<0.05	-	-
Death								
≥120 days	1	1/40	584/15,424	0.65	0.09-4.75	>0.05	-	-
≥ 90 days	4	29/2931	4578/776,907	0.89	0.33-2.82	>0.05	75.8	<0.05
